# What Else Is Happening to the Mirror Neurons?—A Bibliometric Analysis of Mirror Neuron Research Trends and Future Directions (1996–2024)

**DOI:** 10.1002/brb3.70486

**Published:** 2025-04-10

**Authors:** Yangyang Sun, Ningyao Yu, Guanchu Chen, Tongwei Liu, Shengjun Wen, Wei Chen

**Affiliations:** ^1^ Center for Brain, Mind and Education Shaoxing University Shaoxing China; ^2^ Faculty of Education Universiti Kebangsaan Malaysia Bangi Malaysia; ^3^ Department of Psychology Shaoxing University Shaoxing China; ^4^ Department of Philosophy Shanghai Normal University Shanghai China; ^5^ Guangzhou Institutes of Biomedicine and Health Chinese Academy of Sciences Guangzhou China; ^6^ Interdisciplinary Center for Philosophy and Cognitive Sciences Renmin University of China Beijing China

**Keywords:** bibliometrics, interdisciplinary research, mirror mechanism, mirror neurons, neuroscience

## Abstract

**Background:**

Since its discovery in the late 20th century, research on mirror neurons has become a pivotal area in neuroscience, linked to various cognitive and social functions. This bibliometric analysis explores the research trajectory, key research topics, and future trends in the field of mirror neuron research.

**Methods:**

We searched the Web of Science Core Collection (WoSCC) database for publications from 1996 to 2024 on mirror neuron research. Statistical and visualization analyses were performed using CiteSpace and VOSviewer.

**Results:**

Publication output on mirror neurons peaked in 2013 and remained active. High‐impact journals such as *Science, Brain, Neuron, PNAS*, and *NeuroImage* frequently feature findings on the mirror neuron system, including its distribution, neural coding, and roles in intention understanding, affective empathy, motor learning, autism, and neurological disorders. Keyword clustering reveals major directions in cognitive neuroscience, motor neuroscience, and neurostimulation, whereas burst detection underscores the emerging significance of brain‐computer interfaces (BCIs). Research methodologies have been evolving from traditional electrophysiological recordings to advanced techniques such as functional magnetic resonance imaging, transcranial magnetic stimulation, and BCIs, highlighting a dynamic, multidisciplinary progression.

**Conclusions:**

This study identifies key areas associated with mirror neurons and anticipates that future work will integrate findings with artificial intelligence, clinical interventions, and novel neuroimaging techniques, providing new perspectives on complex socio‐cognitive issues and their applications in both basic science and clinical practice.

## Graphical abstract

We conducted a bibliometric analysis of mirror neuron research to examine global publication trends, methodological advancements, and emerging applications. Our findings highlight robust international collaborations, advanced imaging, neurotechnological and immersive technologies, and the translational potential of mirror neuron studies for understanding social cognition and enhancing clinical interventions.

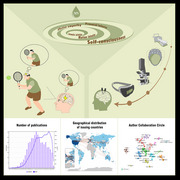

Normal science does not aim at novelties of fact or theory, and when successful finds none. Yet scientific revolutions… require scientists to exhibit both an immersion in tradition and a willingness to destroy it.

Kuhn (1962, 52)

## 1 Introduction

Mirror neurons were a groundbreaking discovery in the early 1990s, profoundly impacting the theoretical and practical foundations across multiple disciplines, including cognitive science (Gallese and Goldman 1998), psychology (Grafton 2009), biology (Tramacere et al., 2017), linguistics (Fogassi and Ferrari, 2007), esthetics (Freedberg and Gallese, 2007), artificial intelligence (Sobhani et al., 2023), sociology (Lizardo, 2007), and anthropology (Arbib, 2011). Extending beyond a simple biological function, these neurons—originally found in the premotor and parietal cortices of the rhesus monkey brain—are activated when an individual performs or observes similar goal‐directed actions. This activation is believed to facilitate a direct understanding (Gallese, 2007; Gallese and Sinigaglia, 2011) or “understanding from the inside” of others’ actions, intentions, and emotions (Rizzolatti and Sinigaglia, 2016, 2023), seemingly mirroring the other person's behavior.

Research on mirror neurons focuses not only on their roles in social cognition, language acquisition, action understanding, and imitation learning but also explores their links to neurological disorders, including autism and Parkinson's disease, aiming to develop novel treatment strategies. The identification of mirror neurons in psychology and neuroscience provides crucial cross‐disciplinary insights, particularly regarding the biological underpinnings of human empathy, enriching fields such as robotics and artificial intelligence. These advances also inspire the design of interactive systems capable of understanding and simulating human behavior more effectively, thereby deepening our comprehension of self‐awareness, cultural inheritance, and social interaction.

Subsequent research has revealed mirroring properties in various human brain regions, including the insula, cingulate gyrus, hippocampal cortex, and cerebellum (Bonini and Ferrari, 2017; Bonini et al., 2022). This finding significantly deepens our understanding of these areas, aided by technological advancements. The evolution from initial electrophysiological recordings to more advanced techniques like positron emission tomography (PET), transcranial magnetic stimulation (TMS), functional magnetic resonance imaging (fMRI), electroencephalogram (EEG), magnetoencephalography, and functional near‐infrared spectroscopy (fNIRS), along with newer methods, such as fiber photometry, drug manipulation, and cortico‐cortical paired associative stimulation, has enhanced insights into their functions and mechanisms.

Ramachandran likened the impact of mirror neurons on psychology to the epochal significance of DNA for biology, asserting, “They will provide a unifying framework and help explain a host of mental abilities that have hitherto remained mysterious and inaccessible to experiments” (Ramachandran, 2000). He suggested that mirror neurons were instrumental not only in enhancing our comprehension of the world but also in shaping our social nature. This perspective sparked further research on mirror neurons over the following 15 years. However, this rapid increase in the number of studies also generated considerable controversy. Reflecting on the role of mirror neurons, some researchers argue, “We will get reliable information about the function of MNs only by applying an approach based on developmental history, system‐level theory, and rigorous experimentation” (Cook et al., 2014). According to Heyes and Catmur (2022), in their study *What Happened to Mirror Neurons?*, “Measured by number of academic publications, interest in mirror neurons peaked 2 years later in 2013 and then began to decline…the mirror‐neuron ‘brand’ is losing its appeal.” Conversely, Bonini et al. (2022) articulate a different view: “The propulsive drive of MNs is not extinguishing but evolving.” In fact, recent statistics from the Web of Science show that the number of publications on mirror neuron research has exceeded 5700, and the citing articles number over 100,000 in total. A Google Scholar search for “mirror neuron” has yielded 58,000 hits (compared to 11,000 in 2014, when mirror neuron research showed signs of decline). This implies that mirror neuron research remains a vibrant topic.

Current qualitative and quantitative studies based on systematic reviews and meta‐analyses have not provided a comprehensive overview of this field. Considering that bibliometrics involves a quantitative evaluation of scientific knowledge's structure through citation analysis (Sabe et al., 2023), which, in combination with systematic mapping, can visualize the evolution of knowledge in the field of mirror neurons, we conducted a comprehensive scientometric analysis. Our study aims to: (1) examine the literature cluster related to mirror neurons, providing a comprehensive overview of the field's development across the last three decades, and (2) analyze changes in the focus and evolutionary trends of mirror neuron research, identifying core research topics and future directions.

## 2 Data Sources and Analysis Methods

### 2.1 Data Collection and Screening

**FIGURE 1:**
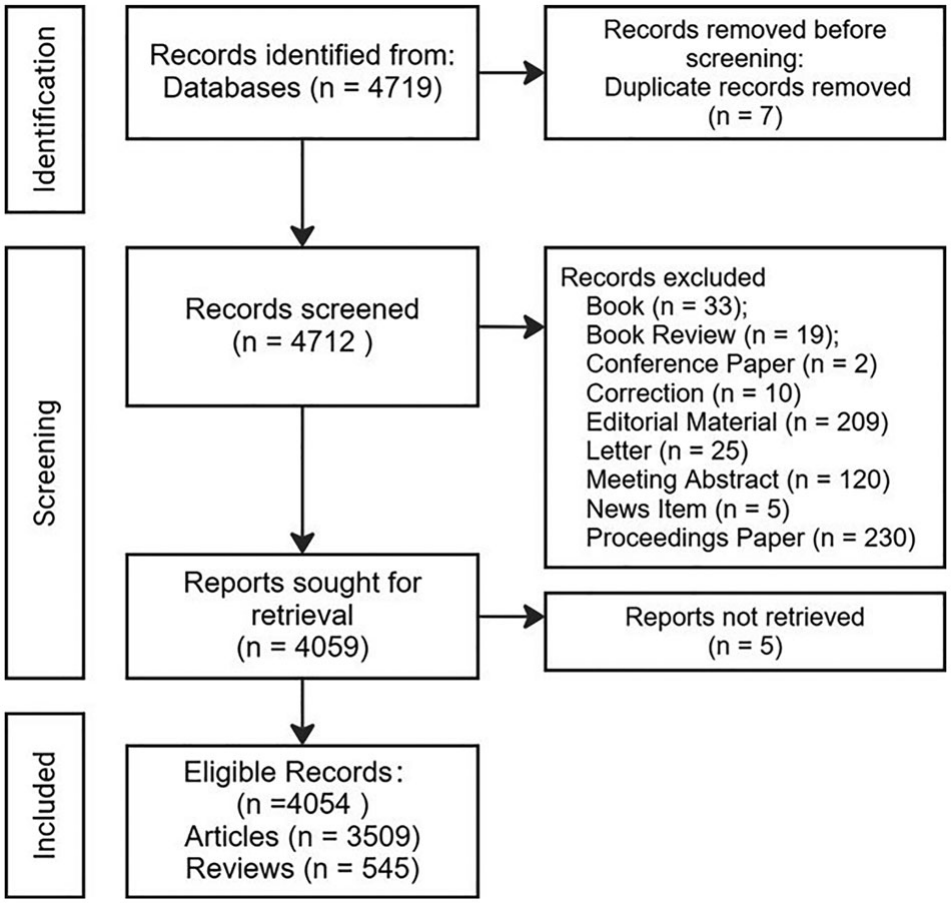
PRISMA study flow chart.

The data utilized in this study were sourced from the Web of Science Core Collection (WoSCC). The search strategy employed the following query: TS = (“mirror neuron*”). Publications such as books and book reviews, corrections, editorial materials, letters, conference papers, news items, and proceedings papers were excluded to ensure the consistency and accuracy of the collected information. Redundant studies were removed, retaining a total of 4054 studies for visualization analysis. The time frame for publication dates ranged from January 1, 1996, to December 31, 2024. The analysis primarily relied on metadata provided for each publication in the WoSCC database, including the country or region of origin, the affiliated institution, the journal in which the work was published, the journal's impact factor, the references cited, the publication year, the total number of citations, the list of authors, and keywords associated with the publication. The retrieved literature was saved in plain text format and exported as a complete record along with the cited references. Three reviewers independently screened the publications and selected those deemed applicable based on their titles and abstracts. Any differing opinions were resolved through discussion. Figure 1 presents the screening process.

### 2.2 Data Visualization and Analysis

We analyzed the dataset using VOSviewer (version 1.6.20) and CiteSpace (version 6.2.R6), tools designed for visualizing and analyzing bibliometric networks. VOSviewer facilitated the analysis of collaboration networks, whereas CiteSpace focused on identifying trends and thematic developments.

Using VOSviewer, we conducted a collaboration network analysis of countries and authors. Node sizes represented publication volumes, colors indicated clusters with relatively strong correlations, and the proximity between nodes reflected the strength of their relationships (van Eck and Waltman, 2014). CiteSpace was then applied to uncover insights into institutional and journal contributions, co‐cited references, and keyword usage. Network analyses examined institutions, journals, co‐cited references, and keywords, with node sizes reflecting publication volumes for institutions and journals, and keyword node sizes indicating their frequency. In addition, citation bursts in co‐cited references and keywords were analyzed to identify topics experiencing sudden increases in attention during specific periods. A timeline view of keyword clusters was used to map the developmental trajectory and temporal distribution of research themes, with clusters arranged horizontally along a time axis and vertically by size, from largest to smallest. Large or red nodes represented higher frequencies or stronger burst intensities (Chen, 2017). We extracted 9 clusters from the 11 keyword clusters, excluding two clusters named “mirror neurons” and “mirror neuron systems.” We believe that these two clusters may lead to confusion in the analysis of research hotspots, as they likely represent broader research areas and encompass themes from other clusters.

## 3 Results

**FIGURE 2:**
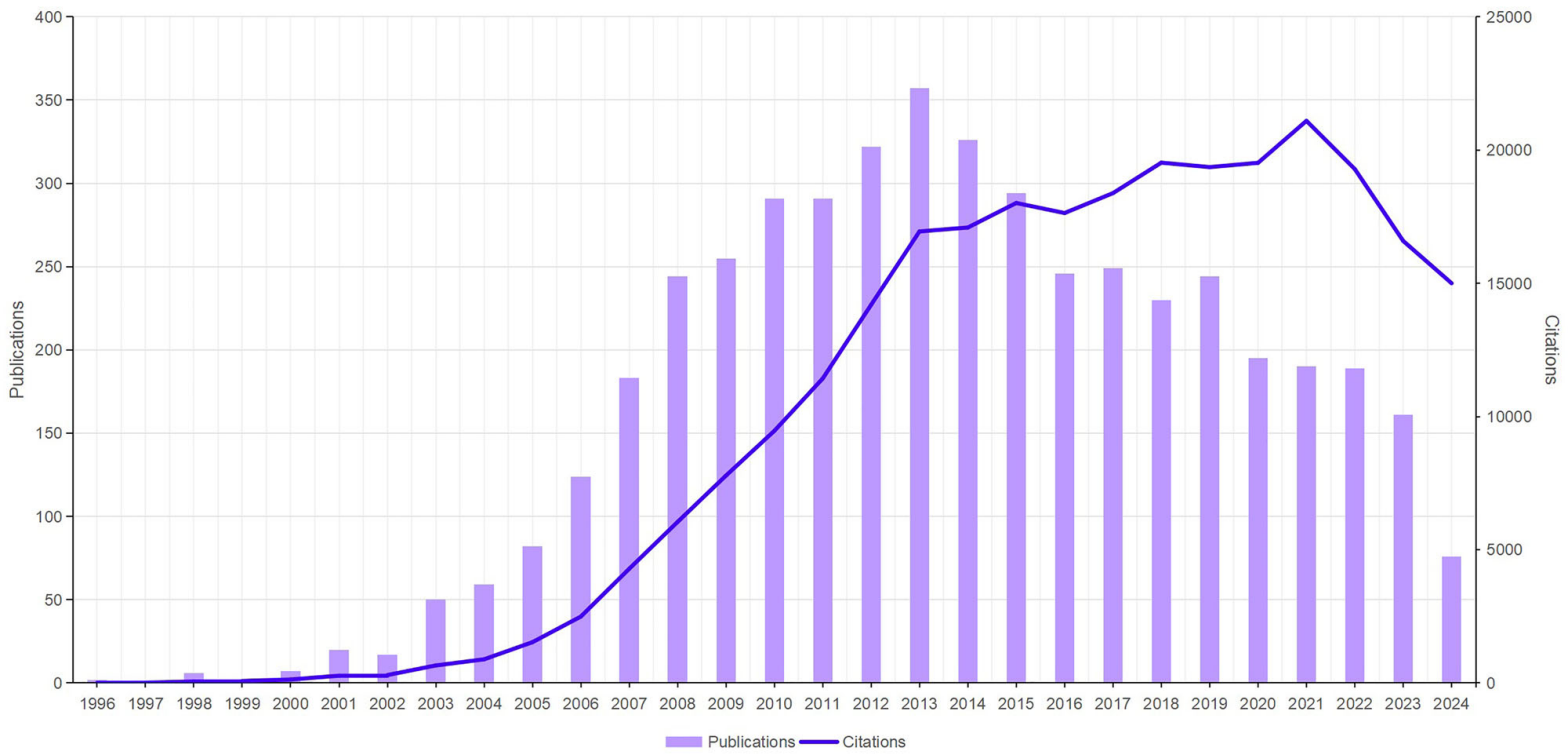
Number of publications and citation frequency in mirror neuron research.

Figure 2 shows the trends in the research results on mirror neurons over the last 30 years. A historical peak was reached in 2013 with 307 articles published. Overall, the frequency of article citations exhibited an upward trend; of note, there was an exponential increase until 2013, followed by a flat rate of growth and a downward trend starting in 2021. Despite the decline in the number of published articles, the depth and impact of research continue to grow. This phenomenon may reveal the intensification of research depth and ongoing advancement in scientific understanding. For example, advanced neuroimaging techniques have enabled researchers to elucidate complex mechanisms of mirror neuron action in social cognition and imitative learning, guiding mirror neuron research toward a more refined and interdisciplinary approach. Advanced neuroimaging techniques, such as fMRI for real‐time brain activity monitoring and TMS for targeted neural modulation, provide powerful experimental tools to validate the mirror neuron hypothesis. These technological advancements enhance the reliability of the research results and promote interdisciplinary cooperation, closely linking knowledge from neuroscience, psychology, computer science, and other fields. Furthermore, numerous studies addressing the association between mirror neuron dysfunction and various neuropsychiatric conditions (e.g., social anxiety disorders and autism spectrum disorders [ASDs]) have emerged since 2013. These studies have sought to uncover the impact of mirror neuron system (MNS) dysregulation on social interaction capabilities, providing a theoretical basis for the development of new therapeutic interventions.

### 3.1 Analysis of Countries and Institutions

**FIGURE 3:**
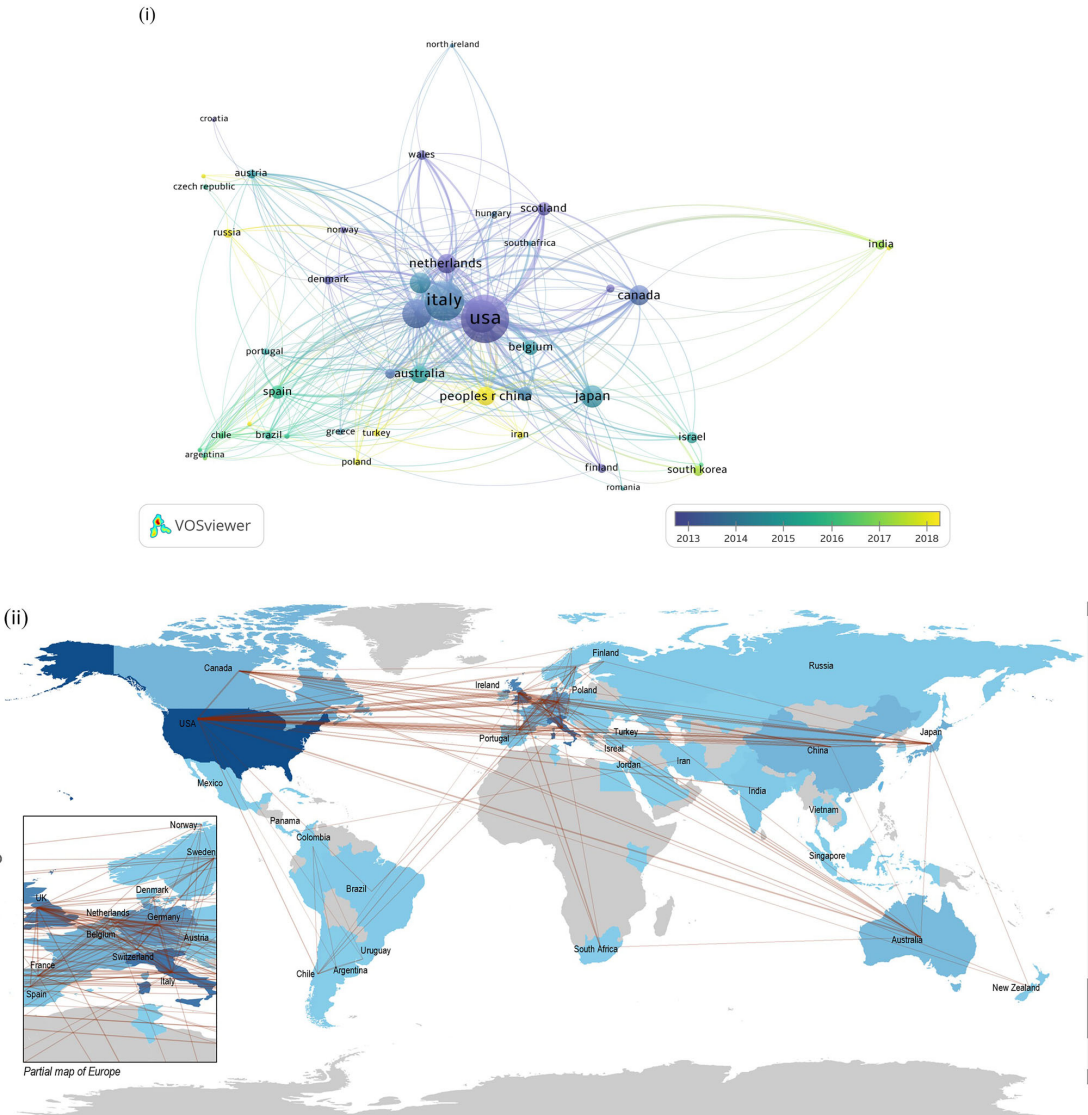
Geographical distribution of publications in mirror neuron research. (i) The intensity and temporal changes of cooperation between countries. (ii) The level of activity and geographical distribution of countries (the deeper the color, the more active the country is in this field of research).

These publications primarily originated from 70 different countries and regions, encompassing 581 research institutions. Figure 3 shows the characteristics of the geographical distribution and international collaborations. Overall, European and North American countries showed higher research activities on mirror neurons. The United States had the highest output (1151, 28.39%), demonstrating its research capacity and influence in this domain, followed by Italy (765, 18.87%), England (545, 13.44%), and Germany (439, 10.83%); all exhibited close collaborative relationships. Table 1 presents the 10 countries and regions with the highest research productivity.

**TABLE 1:** Top 10 countries by number of publications in mirror neuron research.

Rank	Countries	Article counts	Proportion (%)
1	The United States	1151	28.39
2	Italy	765	18.87
3	England	545	13.44
4	Germany	439	10.83
5	France	236	5.82
6	Japan	235	5.80
7	Canada	216	5.33
8	The Netherlands	200	4.93
9	China	181	4.46
10	Australia	168	4.14

The most productive institution was the University of Parma (222), followed by University College London (115), University of California, San Diego (80), and University of California, Los Angeles (78). Table 2 presents the 10 institutions with the highest productivity.

**TABLE 2:** Top 10 institutions by number of publications in mirror neuron research.

Rank	Institutions	Countries	Article counts
1	University of Parma	Italy	222
2	University College London	England	115
3	University of California, San Diego	The United States	80
4	University of California, Los Angeles	The United States	78
5	Radboud University Nijmegen	The Netherlands	68
6	University of Southern California	The United States	66
7	Harvard University	The United States	65
8	University of Oxford	England	58
9	Max Planck Institute for Human Cognitive and Brain Sciences	Germany	47
10	University of Padua	Italy	46

### 3.2 Journal Analysis

A co‐cited journal refers to a journal frequently cited alongside other literature, indicating a close relationship between scholarly sources; the more frequently two articles are co‐cited, the higher their degree of closeness. Of the 1357 journals related to mirror neurons, 62 journals were co‐cited in excess of 500 times, whereas 32 journals exceeded 1000 co‐citations. Notably, as delineated in Table 3, 10 journals were co‐cited more than 2100 times, underscoring their pivotal role in the ongoing scholarly debates. Leading this group, *NeuroImage* was the most co‐cited journal (2879), followed by *Trends in Cognitive Sciences* (2547) and *Science* (2498).

**TABLE 3:** Top 10 co‐cited journals in mirror neuron research.

Rank	Co‐cited journal	Citations	IF (2023)	JCI (2023)	JCR (2023)
1	NeuroImage	2879	4.7	1.49	Q1
2	Trends in Cognitive Sciences	2547	16.7	2.07	Q1
3	Science	2498	44.8	9.90	Q1
4	Neuropsychologia	2360	2.0	0.75	Q3
5	Brain	2328	11.9	3.35	Q1
6	Journal of Neuroscience	2270	4.4	1.32	Q1
7	Proceedings of The National Academy of Sciences of the United States	2256	9.4	2.40	Q1
8	Nature Reviews Neuroscience	2233	28.7	4.29	Q1
9	Experimental Brain Research	2192	1.7	0.45	Q4
10	Cerebral Cortex	2130	2.9	0.93	Q2

### 3.3 Authors Analysis

Co‐cited authors were cited together in one study, indicating that their research is related. An analysis of these authors can reveal influential experts in the field (Table 4). A total of 1263 co‐cited authors were identified, with four experts exceeding 1000 citations: Rizzolatti (2938), Gallese (2241), Iacoboni (1829), and Buccino (1265).

**TABLE 4:** Top 10 co‐cited authors in mirror neuron research.

Rank	Co‐cited authors	Citations
1	Giacomo Rizzolatti	2938
2	Vittorio Gallese	2241
3	Marco Iacoboni	1829
4	Giovanni Buccino	1265
5	Giuseppe Di Pellegrino	953
6	Luciano Fadiga	950
7	Jean Decety	857
8	Christian Keysers	809
9	Leonardo Fogassi	786
10	James Kilner	749

**FIGURE 4:**
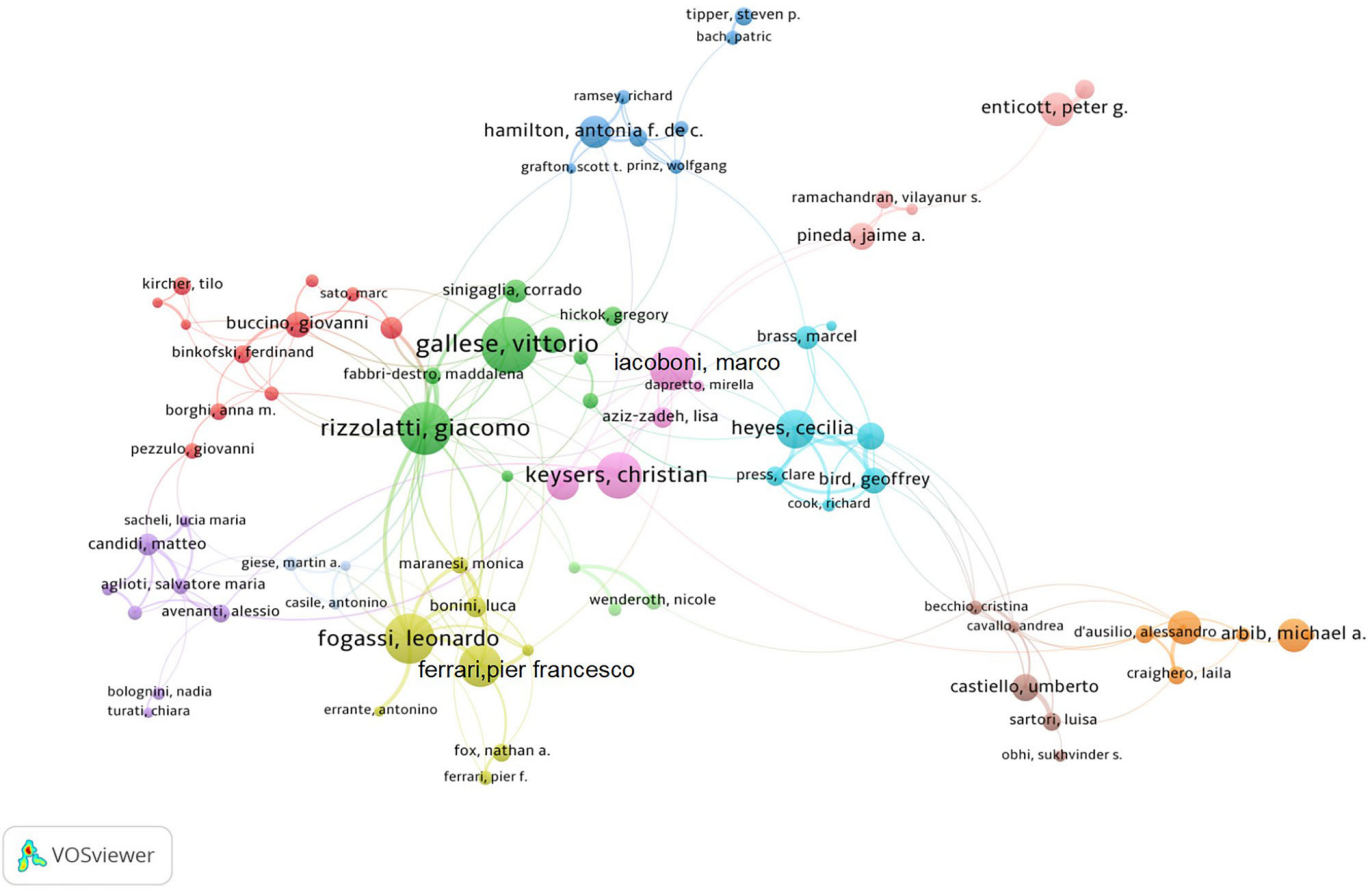
Author collaboration in mirror neuron research.

As per the author collaboration map provided by VOSviewer, scientists collaborate closely on diverse and intertwined research questions. As shown in Figure 4, the green, yellow, and pink collaboration circles consist primarily of authors from the University of Parma, Italy. The green collaboration circle is represented by Rizzolatti and Gallese. Rizzolatti's research focuses on neuroscience and cognitive science, emphasizing the neural mechanisms underlying higher cognitive functions. He applies various methodologies, including the examination of single‐neuron activity in nonhuman primates and the use of brain imaging techniques in humans, to uncover these underlying mechanisms. His work integrates themes such as the motor system and cognitive processes. Gallese's research centers on neurophysiology, cognitive neuroscience, and the philosophy of mind, particularly embodied simulation theory and mirror neuron studies, addressing issues related to action understanding, empathy, language, mind reading, and esthetic experience.

The yellow collaboration circle includes Fogassi and Ferrari. As a codiscoverer of mirror neurons, Fogassi emphasizes the cognitive characteristics of the motor system by studying both primates and humans. Ferrari investigates the neural basis of social behavior and emotion, including the role of mirror neurons in emotional communication and intention, focusing on how the visual system processes motion information and integrates visual and tactile perceptions. The pink collaboration circle includes Keysers from the Netherlands Institute for Neuroscience and Iacoboni from the University of California, Los Angeles. Keysers's research encompasses social neuroscience, neuroimaging, and electrophysiology, focusing on the neural bases of action understanding, empathy, and emotional contagion. Iacoboni focuses on the use of brain imaging (primarily fMRI) and TMS and tDCS to study human system neuroscience, with an interest in imitation, empathy, neurobiological mechanisms, and interventions related to neuropsychiatric disorders.

The light blue and dark blue collaboration circles primarily consist of researchers from UK institutions. Heyes, from the University of Oxford, engages in the theoretical life sciences by exploring the neurocognitive mechanisms of cultural transmission, including social learning, imitation, mirror neurons, and mind reading. Bird, from University College London, studies social cognition and emotional processing, as well as abnormalities in mental disorders, such as autism. Hamilton, also from University College London, focuses on how people understand the mental states of others and the neural basis of this understanding in social interactions. Hamilton employs various methods, including fMRI and EEG, to explore individual differences in theory of mind among individuals and correlate these differences with their social interactions.

The two collaboration circles exhibit some interaction; the circles in green, yellow, and pink provide a solid framework for understanding the biophysical foundations of mirror neurons, whereas the light blue and dark blue circles enhance our comprehension of social cognitive functions. This interdisciplinary dialog deepens our understanding of mirror neurons, opening up new avenues and boundaries for future research into the treatment of related neuropsychiatric disorders and the enhancement of the social interaction capabilities of artificial intelligence.

### 3.4 References Analysis

References that are co‐cited appear together in one or more scholarly articles, indicating a close relationship between the cited works. Table 5 lists the 10 most frequently cited references. Among these, “The mirror‐neuron system” published in 2004 in the *Annual Review of Neuroscience* by Rizzolatti and Craighero was the most cited, offering a systematic overview of the research conducted by Rizzolatti's group. Three further publications on mirror mechanisms from the same group provided critical insights into the functional understanding of mirror neurons.

**TABLE 5:** Top 10 co‐cited references in mirror neuron research.

Rank	Article title	Authors	Year	Journal	Citations
1	The Mirror‐Neuron System	Rizzolatti, G; Craighero, L	2004	*Annual Review of Neuroscience*	734
2	Parietal Lobe: From Action Organization to Intention Understanding	Fogassi, L; Ferrari, PF; (…); Rizzolatti, G	2005	*Science*	364
3	Grasping the Intentions of Others with One's Own Mirror Neuron System	Iacoboni, M; Molnar‐Szakacs, I; (…); Rizzolatti, G	2005	*PLoS Biology*	337
4	The Functional Role of the Parieto‐Frontal Mirror Circuit: Interpretations and Misinterpretations	Rizzolatti, G; Sinigaglia, C	2010	*Nature Reviews Neuroscience*	331
5	Understanding Emotions in Others: Mirror Neuron Dysfunction in Children with Autism Spectrum Disorders	Dapretto, M; Davies, MS; (…); Iacoboni, M	2006	*Nature Neuroscience*	283
6	Action Observation Activates Premotor and Parietal Areas in A Somatotopic Manner: An FMRI Study	Buccino, G; Binkofski, F; (…); Freund, HJ	2001	*European Journal of Neuroscience*	281
7	The Mirror Neuron System and the Consequences of its Dysfunction	Iacoboni, M; Dapretto, M	2006	*Nature Reviews Neuroscience*	280
8	Single‐Neuron Responses in Humans during Execution and Observation of Actions	Mukamel, R; Ekstrom, AD; (…); Fried, I	2010	*Current Biology*	271
9	Hearing Sounds, Understanding Actions: Action Representation in Mirror Neurons	Kohler, E; Keysers, C; (…); Rizzolatti, G	2002	*Science*	260
10	Neurophysiological Mechanisms Underlying the Understanding and Imitation of Action	Rizzolatti, G; Fogassi, L; Gallese, V	2001	*Nature Reviews Neuroscience*	251

**FIGURE 5:**
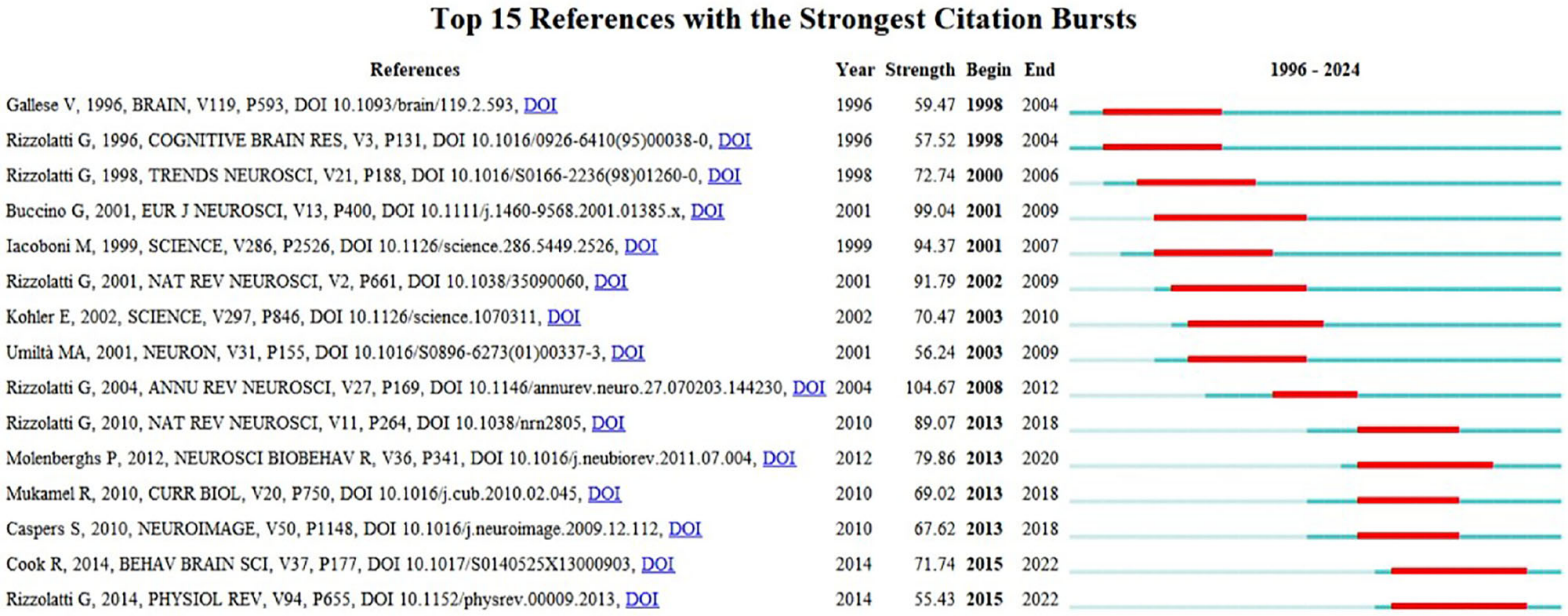
Top 15 references with the strongest citation bursts in mirror neuron research.

Reference bursting describes a surge in the citation frequency of certain references within a specific time span, reflecting a corresponding change in research focus over time. As shown in Figure 5, many highly cited works originated from the pioneering research of Rizzolatti's team, reflecting the substantial impact of their foundational studies on mirror neurons. Since the introduction of the concept of mirror neurons, the team has elucidated the neurobiological basis for understanding how humans and other animals learn actions by observation (Rizzolatti, Fadiga, Gallese, et al., 1996). Their influential literature review published in 2004 synthesized evidence that mirror neurons were found in the monkey brain and were similarly distributed in the human brain and highlighted the link between mirror neuron activity and multiple regions of the brain, including the left inferior frontal gyrus and the right anterior parietal region (Rizzolatti and Craighero, 2004). Rizzolatti, in collaboration with Sinigaglia, published a critical and constructive literature review on the function of the mirror circuit in enhancing the capacity for understanding and imitating observed actions by connecting the parietal and frontal regions of the brain (Rizzolatti and Sinigaglia, 2010). Published at a critical time in the early years of this century, this article clarified previous oversimplifications of the direct mapping theory of mirror neurons and highlighted the system's dynamic flexibility in complex cognitive and social interactions. Since then, the function of mirror neurons has been increasingly discussed in social cognition, affective empathy, and even language understanding. Four years later, Rizzolatti's team continued to consolidate their progress, offering new insights into the motor system for cognitive and behavioral understanding (Rizzolatti et al., 2014). Notably, the researchers emphasized the paradigm shift introduced by mirror mechanisms in cognitive neuroscience and psychology, thereby helping identify multiple research directions for future studies.

### 3.5 Keywords Analysis

**FIGURE 6:**
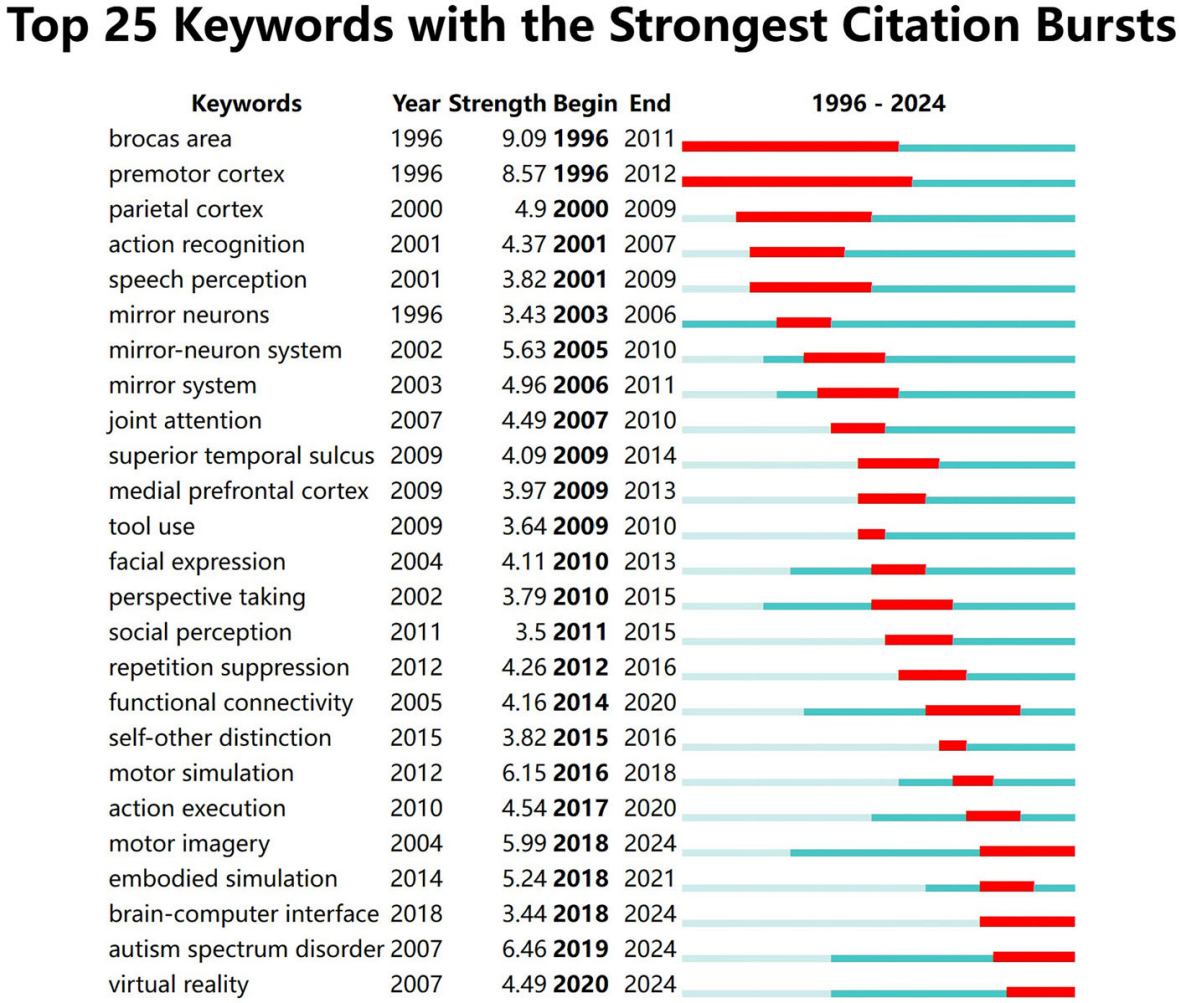
Top 25 keywords with the strongest citation bursts in mirror neuron research.

A keyword analysis highlights the hotspots and emerging trends. Table 6 illustrates the 36 high‐frequency keywords, each with a frequency of more than 20. The keyword “action observation (AO)” appeared 334 times, “social cognition” appeared 193 times, “theory of mind” appeared 148 times, and “transcranial magnetic stimulation” appeared 121 times, followed by “ASD” (94), “action understanding” (87), “embodied cognition” (85), “motor imagery (MI)” (67), “mu rhythm” (63), and “premotor cortex” (61). Analysis of the keyword bursts revealed that “brain‐computer interface (BCI),” “autism spectrum disorder,” and “virtual reality (VR)” exhibited the most recent keyword bursts, which coincides with current research trends of general interest (Figure 6).

**TABLE 6:** High‐frequency keywords in mirror neuron research.

Rank	Count	Year	Keywords	Rank	Count	Year	Keywords
1	1415	1999	Mirror neuron (system)	19	39	2002	Motor system
2	334	1999	Action observation	20	37	2009	Joint action
3	193	2006	Social cognition	21	36	2007	Virtual reality
4	148	1999	Theory of mind	22	35	2009	Social neuroscience
5	121	2001	Transcranial magnetic stimulation	23	33	2003	Motor control
6	94	2007	Autism spectrum disorder	24	30	2010	Mu suppression
7	87	2004	Action understanding	25	30	2006	Motor cortex
8	85	2003	Embodied cognition	26	29	1996	Broca's area
9	67	2004	Motor imagery	27	28	2006	Motor learning
10	63	2004	Mu rhythm	28	25	2005	Functional connectivity
11	61	1996	Premotor cortex	29	24	2011	Predictive coding
12	58	2006	Functional magnetic resonance imaging	30	24	2014	Embodied simulation
13	55	2009	Social interaction	31	24	2011	Action observation network
14	52	2004	Biological motion	32	22	2007	Action prediction
15	49	2008	Motor resonance	33	22	2010	Action execution
16	42	2009	Action perception	34	21	2008	Simulation theory
17	42	2007	Automatic imitation	35	20	1999	Primary motor cortex
18	42	2003	Mirror system	36	20	2003	Inferior frontal gyrus

**FIGURE 7:**
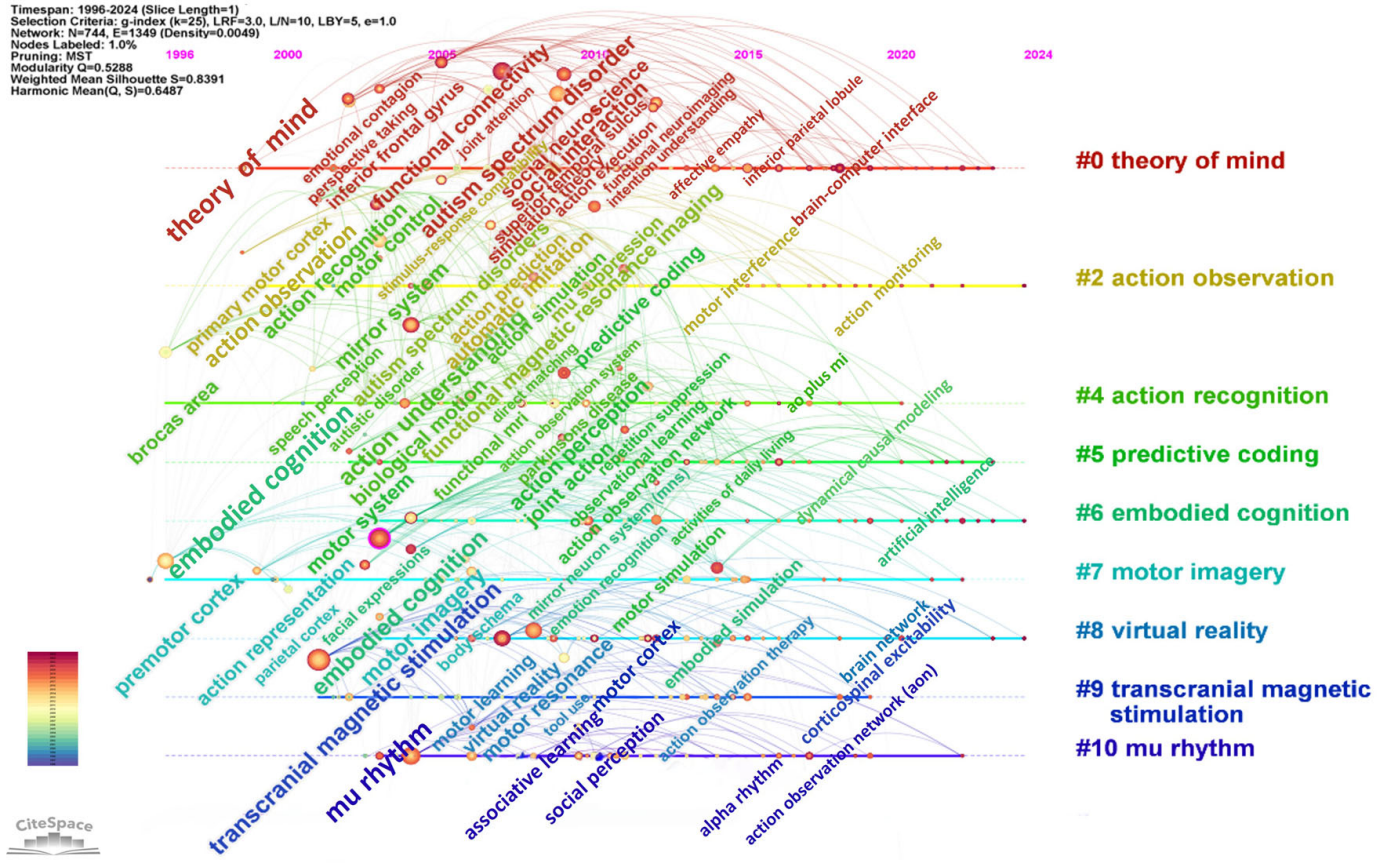
Cluster timeline in mirror neuron research.

The keyword‐clustering timeline identifies three categories of hot topics (Figure 7). (1) Three clusters were associated with research in psychology and cognitive neuroscience (#0 theory of mind, #5 predictive coding, #6 embodied cognition), which explored the function of mirror neurons in comprehending the behavior of others, predicting the outcome of behaviors, and constructing complex cognitions through bodily experiences. A total of 208 keywords were used, including terms such as “autism spectrum disorder,” “social interaction,” “social neuroscience,” “functional connectivity,” “action perception,” “joint action,” “embodied simulation,” and “emotion recognition.” (2) Four clusters were related to research in the field of motor neuroscience (#2 AO, #4 action recognition, #7 MI, and #10 mu rhythm), which explored how motor neuroscience could be used to understand and control movement by integrating multiple modalities of perception, imagination, observation, and recognition. There were 192 keywords, including “premotor cortex,” “motor system,” “action representation,” “associative learning,” “facial mimicry,” “automatic imitation,” “Broca's area,” and “fMRI.” (3) Two clusters involved research in the area of neurostimulation and technology applications (#8 VR and #9 TMS), exploring the potential of neurostimulation and VR technologies for applications in sports rehabilitation and neurological remodeling (particularly in the context of neurological injuries and movement disorders). A total of 71 keywords were used, including “motor resonance,” “motor learning,” “action observation treatment,” “tool use,” “action observation therapy,” and “cerebral palsy.”

**FIGURE 8:**
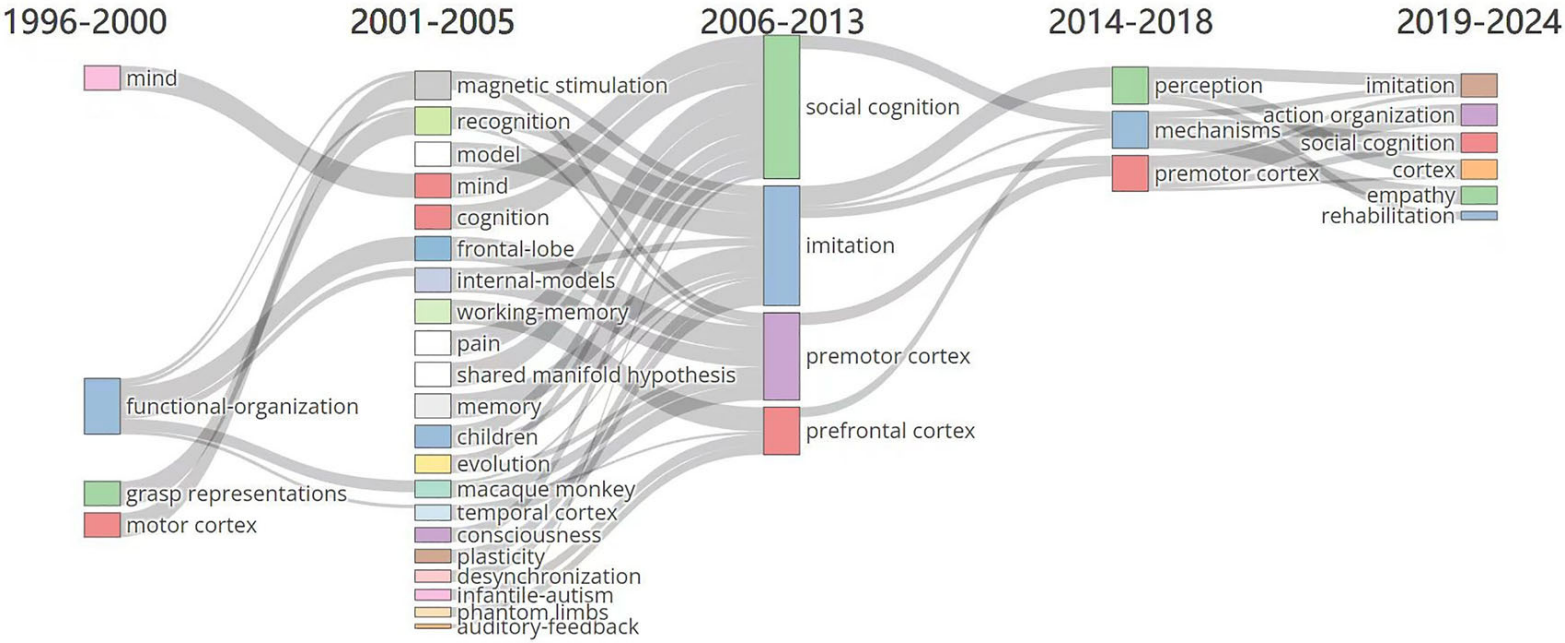
Evolution of keywords in mirror neuron research.

A Sankey diagram shows the flow and transfer relationships of flow, energy, and resources among different stages or components. Figure 8 shows the evolutionary relationships of the main keywords used in mirror neuron research. Early research on mirror neurons attempted to apply this neurobiological discovery across various fields to explain a range of problems. Since 2013, mirror neuron research has declined in popularity, with many studies opting to use alternatives such as “AO network” instead of the term “mirror neurons.” Additionally, the scope of mirror neurons as a general explanatory framework has narrowed with recent findings refining their explanatory power (Dong et al., 2025).

## 4 Discussion

### 4.1 Developmental Trajectory and Theoretical Debates

Over the last 30 years, research on mirror neurons has undergone notable shifts, moving from early discoveries to interdisciplinary expansions. Mirror neurons have offered critical insights into intention understanding, affective empathy, motor learning, and various neurological disorders. This study not only reviews the scientific trajectory of mirror neuron research but also examines key research topics and future trends in this domain.

Early progress in this domain was spearheaded by Rizzolatti et al., who employed high‐precision single‐cell recording techniques in the premotor cortex (area F5) of *Macaca nemestrina* monkeys, revealing the properties of these neurons (di Pellegrino et al., 1992). Subsequent studies extended their reach to other areas of the monkey brain, including the rostral ventral premotor cortex (Keysers et al., 2003), lateral ventral premotor (Ferrari et al., 2005), and superior temporal sulcus (STS) (Jellema et al., 2000). With growing interest in identifying mirror neurons in the human brain, extensive cross‐species research has provided substantial evidence. This burgeoning body of research supports a profound hypothesis that the human mirroring mechanism may engage the entire brain.

However, robust criticisms—notably by Gregory Hickok—have catalyzed a more cautious reappraisal of mirror neurons. Drawing upon a wide array of data from diverse areas, such as animal behavior research, contemporary neuroimaging studies, and investigations of neurological disorders, Hickok reevaluates the limitations of the human mirroring system in action understanding. In his book, *The Myth of Mirror Neurons: The Real Neuroscience of Communication and Cognition*, Hickok emphasizes the necessity of more comprehensively assessing contributions from other brain regions, including the prefrontal cortex and somatosensory areas, and highlights the advantages of predictive coding theory (Hickok, 2014).

Recent studies further challenge the traditional understanding of mirror neurons, indicating the need for a nuanced perspective on their specific roles in motor perception and action understanding. For instance, multivoxel pattern analysis has shown that although mirror neuron brain areas contribute to the low‐level processing of actions, they do not directly engage in high‐level cognitive interpretations of those actions (Wurm and Caramazza, 2019). Research by Pomper et al. (2023) demonstrates that, in most cases, F5 mirror neurons do not encode observed actions using the same neural code as that underlying action execution. These findings suggest that although mirror neurons contribute to the neural representation of actions, their capacity to understand complex cognitive states or the intentions behind those actions is more limited than previously assumed. Moreover, observational learning in real‐world contexts involves complex interactions of multiple cognitive and contextual factors, including action complexity, learning context, and motivation, that surpass the explanatory scope of the MNS alone (Ramsey et al., 2021). Collectively, these challenge the preeminent status that some supporters have attributed to mirror neurons. They are no longer considered the “silver bullet” for solving all issues related to social cognition.

Despite a tempering of the initial excitement, mirror neurons remain a crucial link among action understanding, language development, and even deciphering the pathology of various conditions. Recent studies indicate that the diminishing interest in mirror neurons does not imply that they have ceased to be relevant (Heyes and Catmur, 2022). Rather, it suggests a shift in how these neurons are understood within the scientific community (Dong et al., 2025). Particularly noteworthy is the fact that mirror neuron research has demonstrated a clear trend toward interdisciplinary integration across a wide range of subject areas. This interdisciplinary research is based on multidisciplinary clusters, including the integration of several disciplines drawing on neuroscience, psychology, cognitive science, motor neuroscience, linguistics, and artificial intelligence. The following sections will explore the “ripple effect” of mirror neurons, illustrating how traditional social cognition topics have expanded into new areas and significantly influenced broader fields of psychology and neuroscience. We will also discuss the critical contributions of mirror neurons to understanding actions and facilitating motor learning, emphasizing the importance of this research in developing innovative clinical methodologies. Finally, we will examine how emerging technologies and clinical applications are driving the field forward, highlighting the role of these advancements in shaping the future of neurorehabilitation and treatment modalities.

### 4.2 The “Ripple Effect” of Mirror Neurons

A prominent way to conceptualize the role of mirror neurons in social cognition involves understanding their “ripple effect.” This term describes how insights from neural mechanisms gradually expand to offer novel perspectives for addressing various problems. It has traditionally been argued that inferring mental states is a crucial component of theory of mind. An enduring controversy between “theory‐theory” and “simulation theory” persists regarding the underlying mechanisms of this ability. Building on Rizzolatti's (2001) findings on the function of mirror neurons in monkeys, subsequent research posits the existence of a similar system in the human brain. A growing body of evidence suggests that this MNS underpins imitation learning, offering neuroscientific support for simulation theory (Iacoboni, 2009). This discovery highlights not only conceptual reasoning but also the role of direct imitation in understanding other minds.

Building on this foundation, increasing evidence reveals that social affective processes, such as empathy, language evolution, social learning, emotion recognition, and self‐awareness, are linked to mirror neuron functioning (Corballis, 2010; Keysers et al., 2022; Keysers and Michon, 2024; Whiten and van de Waal, 2017; Yokose et al., 2024). This connection is especially evident in empathy: observations of others’ painful or joyful experiences elicit neural activations overlapping with first‐person experiences (Bekkali et al., 2021; Carr et al., 2003; Plata‐Bello et al., 2023; Tanzer and Weyandt, 2019).

Moreover, studies on mirror neurons in non‐primates also reveal similar mechanisms of empathy. Mirror neurons exist in various vertebrates, including birds, bats, and rodents, and vary in function and distribution (Bonini et al., 2022). In rodent models, for instance, neurons in the anterior cingulate cortex (ACC) were found to be active during both direct experience and observation of conspecifics in pain (Carrillo et al., 2019). This research identified mirror neurons in the emotional system, highlighting the status of the ACC as an empathic response center and suggesting that pain empathy has tunable properties. Similarly, mirror neurons were identified in the hypothalamus of mice, specifically in the ventral medial hypothalamic region, and were strongly correlated with aggression in male mice (Yang et al., 2023). These findings show that mirror neurons contribute to the social cognitive process by comprehending the actions and intentions of others, and they may also impact the transmission of negative behaviors, such as aggression, while imitating and learning positive behaviors (Ferrari et al., 2023).

Extending the discussion on empathy and social behavior, recent research also implicates mirror neuron mechanisms in empathy‐driven prosocial behavior. For example, a study found that human prosocial decision‐making activates brain regions, such as the STS and inferior parietal lobule (IPL), which are instrumental in the perception of biological movements, action understanding, imitation, and the interpretation of motor intentions (Wu and Hong, 2022). Similarly, studies on rodents have demonstrated their capacity to perceive and appropriately react to the emotions of conspecifics. This ability is mediated by emotional mirror neurons and specialized neural circuits, which support pro‐social behavior to some extent (Keysers et al., 2022).

Finally, a recent study further consolidates these insights by revealing mirror‐induced self‐directed behavior in mice. In this study, mice were observed integrating visual and tactile information. This behavioral process required social experience and mirror habituation and was accompanied by neuronal activation in the hippocampus, particularly in the ventral hippocampal vCA1 region (Yokose et al., 2024). These findings suggest a possible correlation among the mirror neuron mechanisms. Overall, these studies offer significant revelations regarding the evolutionary origins and neural foundations of self‐awareness and advanced social cognition in humans.

### 4.3 Action Understanding and Motor Learning Mechanisms

In the domain of motor control, the most widely recognized contribution of mirror neurons is the “observation‐execution matching” mechanism. The cerebral cortex is activated when observing the actions of others and overlaps with areas activated when the individual performs the actions themselves. This mechanism supports internal simulation of observed actions and thus helps in understanding the action (Rizzolatti et al., 2014). In addition, these neurons significantly contribute to essential neurophysiological processes, such as MI, motor learning, and relearning, by unifying action perception and action execution through an observation‐execution matching mechanism.

The motor learning of complex skills is enhanced by MI and the AO. Investigations into the properties of parieto‐frontal mirror neurons suggest that these neurons encode observed motor acts with a notable degree of generality (Rizzolatti and Sinigaglia, 2010). The MNS, encompassing the ventral premotor and subparietal regions, facilitates motor learning of complex gestures more effectively through AO than through MI (Gatti et al., 2013). At the neurophysiological level, the AO can modulate the excitability of the corticospinal system, particularly the activity of the premotor cortex (Loporto et al., 2011), by increasing the amplitude of motor‐evoked potentials, which can enhance the acquisition of motor gesture learning. Additionally, mirror neurons appear to adapt through training. For example, studies have shown that after short‐term executive training, subjects display enhanced activation of the sensorimotor cortex when observing specific hand movements, indicating a significant effect of such training on the MNS (Brunsdon et al., 2020). Further supporting these findings, researchers have also assessed the correlation between competence and neural function in dancers and nondancers. They noted that mirror neuron regions are more active in dancers than in nondancers when observing dance performances (Calvo‐Merino et al., 2005; Orlandi et al., 2017).

At the same time, an increasing body of research has presented evidence for interconnections between the MNS and the cerebellum. A meta‐analysis by Molenberghs et al. (2012) confirmed the presence of neurons in the cerebellum that exhibit mirroring properties. Specifically, research has shown that the cerebellum plays a critical role in inferring others’ mental states by understanding the sequences of their actions (Van Overwalle et al., 2019). Further studies demonstrate that the cerebellum is involved in the MNS during AO and MI. Moreover, cerebellar injury may impair mirror neuron function, underscoring the cerebellum's critical role in AO and MI processes (Antonioni, Raho et al., 2024). Research also confirms that the cerebellum, basal ganglia, and thalamus all exhibit somatotopically organized activation during action execution, indicating that these subcortical structures may serve as an extended part of the MNS, integral to action understanding and internal simulation (Errante et al., 2023; Ramsey et al., 2021). More recently, research has expanded the critical role of the cerebellum within the MNS. In a complex bimanual action sequences imitation task, Errante et al. (2025) reported that activation in the MNS, prefrontal cortex, and cerebellum predicts participants’ imitation accuracy. These findings indicate that the cerebellum's key role in generating predictive motor representations closely collaborates with core MNS areas, thereby promoting more precise observation and imitation.

This further supports the idea that motor learning depends on the MNS and emphasizes the critical involvement of these subcortical structures, particularly the cerebellum, in facilitating its effective functionality. Future studies need to clarify how the cerebellum and cortical mirror neuron regions collaborate to facilitate learning action sequences. This could involve using neural representational similarity analysis to explore the interactions in action representation between the cerebellum and cortex, as well as employing functional connectivity and brain network modeling to investigate the dynamic neural interactions between these areas.

Nevertheless, debates persist over the scope and limits of mirror neuron function. Experiments, such as those by Catmur et al. (2007), attempting to distinguish between the learning mechanisms within mirror neurons, have indicated that sensorimotor learning influences the initial development of mirror responses; however, it remains to be verified whether this learning is substantial enough to modify the pattern of mirror neuron activity. Likewise, current research has not definitively established a direct involvement of mirror neurons in the processing of actions. Some studies even suggest that mirror neurons are limited to lower levels of processing actions (e.g., recognizing the type of grasping action), and there is a lack of evidence of higher levels of involvement (e.g., inferring the beliefs or intentions of others) (Thompson et al., 2019). The mirror properties within pivotal regions of the motor network have prompted speculation about their influence on neural circuits involved in the planning and execution of actions.

Expanding beyond the basic motor learning, recent studies have begun to explore how the MNS‐cerebellum network supports the complex behaviors essential for human evolution, such as tool use. The core functions of the MNS—observational learning, imitation, and predictive motor control—are critical for acquiring and transmitting tool‐making skills. The cerebellum, in particular, plays a critical role in optimizing the cognitive and social functions required for tool manufacturing. It is involved in predictive control, sequence learning, and internal modeling of actions, which may have facilitated the development of cumulative evolution (Van Overwalle et al., 2022; Vandervert et al., 2024). Within this framework, the cerebellum interacts with subcortical structures such as the basal ganglia and striatum to form an extended network that is distinct from the classical parieto‐frontal mirror circuit, providing the foundation for movement execution and observation (Bonini, 2016; Errante and Fogassi, 2020; Errante et al., 2023). Consequently, the use of tools and their cross‐generational transfer may rely on a broader cortical–subcortical network, not confined to traditional cortical mirror neuron regions. Moreover, the cerebellum, along with the striatum, ventral tegmental area, and prefrontal cortex, plays a pivotal role in the brain's reward mechanisms (Manto et al., 2024), reinforcing behaviors associated with tool use. For example, the cerebellum's ability to anticipate the consequences of actions enables it to foresee the rewards tied to tool use, thereby significantly enhancing the reinforcement of these adaptive behaviors. The interactions between these systems shape the neural basis of human technological evolution, suggesting a broader impact on evolution.

Furthermore, future research is required to obtain robust evidence, with topics from the perspective of cultural evolution presenting substantial research significance. These include the origins and critical periods of sensorimotor experience development that give rise to mirror neurons, the exploration of their variation across cultures, and their potential application in clinical and educational interventions (Heyes and Catmur, 2022).

### 4.4 Technological Advances and Clinical Applications

Rapid advances in technology and methodology have enabled more precise investigations in clinical applications. Initial studies relied heavily on PET (Rizzolatti, Fadiga, Matelli, et al., 1996) and fMRI (Dapretto et al., 2006). Later, stereo‐EEG (Caruana et al., 2017), intracranial EEG (Mukamel et al., 2010; Qin et al., 2023), and fNIRS (Ge et al., 2019) further enriched the methodological arsenal. Researchers can now monitor brain activity more precisely, deepening our understanding of the complexities of the MNS, particularly in applications related to neurorehabilitation. The recent adoption of multimodal/integrated approaches, such as simultaneous EEG‐fNIRS, further enhances this precision by capturing both spatial and temporal dimensions of MNS neural activity. As a result, a variety of rehabilitation strategies and therapies based on mirror neurons have been widely implemented in clinical settings, benefiting greatly from these methodological advancements.

#### 4.4.1 MNS Interventions in ASD and Neurodegenerative Diseases

Against this multifaceted research background, investigators gradually turned their attention to the specific role of mirror neurons in interventions for special populations, notably in ASD. Early MNS‐based interventions have shown improvements in social interaction abilities in children with autism (Calvo‐Merino et al., 2005). Dapretto et al. reported abnormal activation in the pars opercularis of individuals with ASD when processing facial expressions, a finding supported by EEG evidence of disrupted mirror neuron activity (Dapretto et al., 2006; Oberman and Ramachandran, 2007). More recently, the adoption of multimodal/integrated neuroimaging approaches has significantly enhanced the reliability of ASD diagnoses (Abbas et al., 2023; Khan and Katarya, 2025). Although these results underscore the potential of using such technologies to better understand the neurobiological underpinnings of social deficits in ASD, they also suggest that therapeutic approaches targeting mirror neurons could be refined to enhance social interaction skills in ASD populations.

Similarly, the role of the MNS in neurodegenerative diseases has also garnered increasing attention. For instance, patients with behavioral variant frontotemporal dementia (bvFTD) exhibit significant impairments in emotion recognition, closely related to the MNS dysfunction (Jastorff et al., 2016; Weise et al., 2024). Specifically, the ability of bvFTD patients in emotion detection and emotion categorization tasks correlates with the gray matter volume in the left anterior temporal lobe and the left inferior frontal gyrus. Notably, the functional connectivity between these regions is diminished in bvFTD patients, potentially contributing to their behavioral symptoms. These findings reinforce the notion that MNS‐related dysfunction can underlie socio‐emotional deficits. In response, intervention measures such as AO treatment have shown potential. By stimulating the MNS, these measures may improve the social cognition and emotion recognition abilities of patients with bvFTD (Farina et al., 2020). Aligned with ongoing research on mirror neuron interventions, these insights may eventually inform strategies aimed at improving social interaction and emotional empathy in various neuropsychiatric conditions.

#### 4.4.2 Neurorehabilitation Applications of MNS: Mirror Therapy and AO Therapy

Beyond ASD interventions, mirror neurons also show considerable promise across other clinical rehabilitation settings. Mirror therapy and AOT have proven effective in rehabilitating patients with stroke or phantom limb pain, partly by promoting reorganization of motor circuits through mirror neuron activation (Mao et al., 2020). Research by Antonioni, Galluccio et al. (2024) illustrates that event‐related desynchronization during AO serves as an early predictor of recovery in subcortical stroke, emphasizing the value of EEG biomarkers related to mirror neuron activity in enhancing the precision of neurorehabilitation outcomes. Furthermore, recent research indicates that mirror‐gazing can indirectly enhance MI by increasing corticospinal excitability (Iwanami et al., 2023). Meanwhile, TMS studies suggest that even patients with disorders of consciousness may retain residual cognitive processing abilities when observing motion stimuli (Mancuso et al., 2024). Importantly, Boni et al. (2023) have developed a novel protocol that integrates AOT with EEG monitoring, showing promising results in arm motor recovery post‐stroke, particularly benefiting patients with mild to moderate impairments. These findings underscore the versatility of MNS applications in clinical settings and highlight the emerging role of integrated neuroimaging and neurophysiological measures in advancing our understanding and application of neurorehabilitation strategies.

#### 4.4.3 Integration of MNS With Emerging Rehabilitation Technologies: BCI and VR

Alongside traditional clinical interventions, the emergence of BCIs has created new possibilities for applying mirror neuron research in neurorehabilitation. By leveraging the mechanisms of AO and imitation, BCI‐based training protocols can assist patients with impaired motor function to visualize and control robotic limbs or prosthetic devices in real time (Singh et al., 2021; Syrov et al., 2023). Additionally, VR technology has also benefited from mirror neurons. VR systems have demonstrated advantages in the rehabilitation of movement deficits by activating the MNS when observing human actions. The principles of MNS have been integrated into the VR system design (Cameirao et al., 2010), allowing users to enhance immersion and learning outcomes through virtual avatars. For example, a VR climbing experience can simulate the sensation of foot contact with the ground in the real world; it creates a virtual perception of the actual execution of body movements from a first‐person perspective (Maggio et al., 2022). This experience not only facilitates the process of learning by imitation but also improves neuronal activity and connectivity, thereby enhancing neurorehabilitation outcomes. Research has shown that VR therapy based on mirror neurons aids in the upper limb function recovery in patients following stroke (Hao et al., 2023) and improves neurophysiological indicators (Hao et al., 2022). Primavera et al. (2024) have also provided further evidence supporting VR cognitive remediation via activation of the MNS, demonstrating that immersive VR training significantly improves complex cognitive skills in young adults with bipolar disorder. These findings highlight the potential of interventions based on mirror neuron mechanisms in emerging technologies.

Notably, recent studies, such as those by Lanzarini et al. (2025), have observed that certain neurons exhibit higher activity and variability in freely moving contexts. Future research could explore whether mirror neurons exhibit similar selective coding under comparable dynamic conditions, adjusting their neural responses based on changes in context. By revealing the neural strategies that govern natural behaviors, this insight may guide the development of more effective neuromodulation and robot‐assisted neurorehabilitation approaches.

## 5 Conclusion

This article presents an overview of mirror neuron research, which has attracted considerable attention worldwide. Scientists from the United States, Europe, and Asia are leading advancements in this field, and interdisciplinary and cross‐border collaboration have enhanced the depth and scope of the research. Some high‐impact academic journals, such as NeuroImage, frequently publish mirror neuron research and have become a central platform for knowledge exchange in this field. The coding properties of mirror neurons and their roles in social cognition are worthy of attention. The neural mechanisms underlying emotional empathy and understanding others’ intentions establish a theoretical foundation, particularly for the treatment of social disorders such as autism. With advancements in neuromodulation technology and artificial intelligence, direct interventions targeting the MNS have become possible, opening new avenues for clinical treatment. Further analysis of the complex dynamics of the mirror neuron network and its association with consciousness and self‐awareness is an important direction for future research.

In conclusion, this visual analysis summarizes current advancements in mirror neuron research and identifies potential paths for future exploration. It provides a clear scientific overview to guide the exploration of approaches to enhance human self‐perception and social interaction abilities, aiming to translate laboratory‐based research findings to clinical applications.

Several limitations warrant acknowledgment. The primary limitation concerns the concentration of the data sources. Although the Web of Science database is highly regarded in academia, overreliance on a single database may result in the omission of potentially significant data. Future research should utilize additional database resources such as PubMed, Scopus, and preprint platforms to achieve a more comprehensive literature coverage. Second, this study focused on the literature in English language, overlooking research findings reported in non‐English languages. This approach may have inadvertently filtered out culturally specific research or significant contributions from scholars in non‐English‐speaking countries. Considering the global nature of scientific research, future studies should explore strategies for integrating multilingual literature to enhance the universality and applicability of their conclusions. Finally, although this study has endeavored to capture the most recent research developments, given the rapid pace of scientific advancements, the analysis is limited to the most recent data available at the time of writing and does not include newly published findings. Accordingly, readers should be cognizant of potential biases when evaluating the findings of this research. Moreover, although the bibliometric approach effectively identifies overarching research trends, it cannot critically evaluate the methodological rigor of the included studies. This underscores the need for complementary analyses, such as systematic reviews, to offer a more in‐depth assessment of research quality. Future studies are encouraged to address these limitations to enhance our comprehensive understanding of the mechanisms of mirror neurons from diverse perspectives.

## Author Contributions

**Yangyang Sun**: formal analysis, methodology, visualization, writing – review and editing, validation, investigation. **Ningyao Yu**: data curation, formal analysis, visualization, writing – original draft, investigation. **Guanchu Chen**: visualization, writing – original draft, formal analysis, investigation. **Tongwei Liu**: visualization, data curation. **Shengjun Wen**: writing – review and editing, validation. **Wei Chen**: conceptualization, supervision, project administration, writing – review and editing, funding acquisition, validation.

## Conflicts of Interest

The authors declare no conflicts of interest.

### Peer Review

The peer review history for this article is available at https://publons.com/publon/10.1002/brb3.70486.

## Data Availability

The data that support the findings of this study are available from the corresponding author upon reasonable request.
